# Chemical Vapour Deposition Graphene–PMMA Nanolaminates for Flexible Gas Barrier

**DOI:** 10.3390/membranes12060611

**Published:** 2022-06-12

**Authors:** Antonio Baldanza, Maria Giovanna Pastore Carbone, Cosimo Brondi, Anastasios C. Manikas, Giuseppe Mensitieri, Christos Pavlou, Giuseppe Scherillo, Costas Galiotis

**Affiliations:** 1 Department of Chemical, Materials and Production Engineering, University of Naples Federico II, P.le Tecchio 80, 80125 Naples, Italy; antonio.baldanza@unina.it (A.B.); cosimo.brondi@unina.it (C.B.); gscheril@unina.it (G.S.); 2 Institute of Chemical Engineering Sciences, Foundation for Research and Technology—Hellas (FORTH/ICE-HT), 26504 Patras, Greece; mg.pastore@iceht.forth.gr (M.G.P.C.); cpavlou@iceht.forth.gr (C.P.); 3 Department of Chemical Engineering, University of Patras, 26504 Patras, Greece; manikas@chemeng.upatras.gr

**Keywords:** graphene, poly (methyl methacrylate), nanolaminate, barrier properties, carbon dioxide, oxygen, chemical vapour deposition

## Abstract

Successful ways of fully exploiting the excellent structural and multifunctional performance of graphene and related materials are of great scientific and technological interest. New opportunities are provided by the fabrication of a novel class of nanocomposites with a nanolaminate architecture. In this work, by using the iterative lift-off/float-on process combined with wet depositions, we incorporated cm-size graphene monolayers produced via Chemical Vapour Deposition into a poly (methyl methacrylate) (PMMA) matrix with a controlled, alternate-layered structure. The produced nanolaminate shows a significant improvement in mechanical properties, with enhanced stiffness, strength and toughness, with the addition of only 0.06 vol% of graphene. Furthermore, oxygen and carbon dioxide permeability measurements performed at different relative humidity levels, reveal that the addition of graphene leads to significant reduction of permeability, compared to neat PMMA. Overall, we demonstrate that the produced graphene–PMMA nanolaminate surpasses, in terms of gas barrier properties, the traditional discontinuous graphene–particle composites with a similar filler content. Moreover, we found that the gas permeability through the nanocomposites departs from a monotonic decrease as a function of relative humidity, which is instead evident in the case of the pure PMMA nanolaminate. This work suggests the possible use of Chemical Vapour Deposition graphene–polymer nanolaminates as a flexible gas barrier, thus enlarging the spectrum of applications for this novel material.

## 1. Introduction

Two-dimensional (2D) crystals are the most promising materials to date, thanks to their extraordinary physico-chemical properties and their potential to replace conventional ones in several applications [1]. Graphene, for instance, exhibits outstanding mechanical, electronic, optical and barrier properties, and is a promising candidate for many applications, from flexible electronics to composites and coatings [1,2]. The characteristic two-dimensional shape of graphene and related materials triggers these unique properties, which include, among others, the capability to block small penetrant molecules [3]. Excellent results have recently been reported in the literature regarding the mass transport of low-molecular-weight compounds (penetrants) through graphene and other 2D materials [4]. It has been found that pristine, exfoliated graphene is impermeable to several penetrants, including helium [5,6]. In fact, due to the high electronic density of the carbon rings, graphene can repulse atoms and gas molecules as well as water, leading to an intrinsic extremely low permeability of the defect-free layer [7]. However, macroscale sheets of perfect graphene without defects are impossible to obtain by mechanical exfoliation, as this technique only yields micron-sized flakes [1].

Most of the attempts reported in the literature towards practical application of graphene as gas barrier concern the fabrication of nanocomposite membranes or coatings, which are obtained by dispersing liquid or chemically exfoliated graphene flakes and related materials (GRMs)—such as graphene nanoplatelets (GNPs), graphene oxide (GO) and reduced graphene oxide (rGO)—into a polymer matrix [7,8,9]. In principle, a homogeneous dispersion of impermeable graphene nanosheets results in depleted gas permeability of graphene–polymer nanocomposites due to the increased diffusion path of the permeant [10]. Additionally, the barrier properties of the composite depend on the aspect ratio (AR), the loading, the degree of dispersion and the orientation of the graphene nanosheet, as well as on the structure organization of the polymeric chains at the interface with the graphene nanosheets [11]. Overall, the incorporation of GRMs in the form of discontinuous nanosheets into polymer matrices has been found to contribute to significant enhancement of gas barrier properties [7]. However, it is important to note that low loadings of graphene nanosheets can even increase the permeability of polymers to gases [12,13]; on the other hand, nanocomposites with higher GRM loadings that are effective for gas barrier applications may suffer from embrittlement due to poor dispersion of the nanosheets and aggregation phenomena, thus creating structural defects or deteriorating the mechanical properties of the polymer [14].

In general, the typical structure of the discontinuous graphene–polymer composites lacks the precise architecture and 2D nano-porous structure that is characteristic of all-graphene, highly packed membranes [8]. In principle, the optimization of barrier properties can be achieved (with the lowest amount of nanomaterial) by producing films of alternating graphene and polymer layers [15]. In such multilayer membranes, a crucial role is played by the distribution of defects (nanometric holes and/or microcracks) onto the graphene layer, providing prevalent permeation paths to the penetrant molecules in passing through [16,17]. In this regard, it is important to remark that it is not feasible to produce GRM-based nanocomposites that are also multilayer in which the nanosheets are free of defects [18,19]. In fact, according to statistical thermodynamics, mono- and multi-vacancies on the 2D graphene lattice are intrinsically present for entropic reasons at T > 0 K [19]. Moreover, additional defects can be induced in single graphene layers by the manufacturing process [18,19]. The distribution of size and shape of these voids directly governs the permeability mechanism of the graphene nanosheets and, in turn, the barrier property performances as well as the gas permeation selectivity of the nanocomposites [9].

The production of high-quality, continuous, macroscopic 2D materials with controlled layer thickness and reduced number of defects is a growing field that has garnered considerable attention [15] and which has recently provided new opportunities for the preparation of effective composites with unprecedented structural and multifunctional properties. Chemical Vapour Deposition (CVD), for instance, has been widely used to synthesize wafer-scale 2DM and represents a good compromise between quality, quantity, and cost. Recently, some authors have proposed a novel way to incorporate cm-size CVD graphene monolayers into a polymer matrix with a controlled, nanolaminate architecture. The obtained CVD graphene–poly (methyl methacrylate) (PMMA) nanolaminate membranes were found to present enhanced mechanical, electrical and EMI shielding properties, with record values compared to the state-of-the-art [20]. Herein, we demonstrate that the controlled incorporation of CVD graphene into the nanolaminate configuration not only offers improved gas barrier properties compared to the polymer matrix but outperforms traditional discontinuous GRM composites with the same graphene content. In this regard, steady-state permeation measurements were performed with two typical gases of interest (namely, CO_2_ and O_2_) for gas barrier applications, at different levels of relative humidity (R.H.).

## 2. Materials and Methods

### 2.1. Material Preparation

Nanolaminate membranes were produced by iterative transfer of graphene–PMMA (Gr-PMMA) layers using the ‘lift-off/float-on’ process combined with the wet depositions described in [20]. Single-layer graphene was grown on copper foil (JX Nippon Mining & Metals, 35 μm thick, 99.95%) in a commercially available CVD reactor (AIXTRON Black Magic Pro, Herzogenrath, Germany). A commercial solution of polymethyl methacrylate (PMMA) (495 PMMA A6, Microchem Corp., Erlenbach, Switzerland) was adopted for the fabrication of the polymer matrix. Each Gr/-PMMA layer was fabricated by spin coating the PMMA solution on the CVD graphene on Cu foil, at 2000 RPM for 1 min at RT. The copper was then etched away with an aqueous solution of ammonium persulphate (APS) and the remaining Gr-PMMA layer was rinsed with distilled water. By slowly reducing the water level, the floating Gr-PMMA layer was then deposited on another Gr-PMMA layer on a Cu foil, which represents the substrate for subsequent depositions. After each deposition, the multi-layer was firstly dried at 40 °C under vacuum to remove the excess of water and then post-baked at 150 °C for a few minutes to improve adhesion between subsequent layers. The cycle was repeated until the desired number of Gr-PMMA layers was reached (20). At the end, the Cu substrate was etched away in APS solution to release the freestanding Gr-PMMA nanolaminate, then rinsed with water and finally dried at 40 °C under vacuum to remove the excess of water. A similar procedure was adopted to produce a control nanolaminate of neat PMMA, in which bare Cu foils (without graphene) were used as sacrificial substrates.

### 2.2. Thickness Measurements for the Evaluation of Graphene Volume Fraction

The thickness of the single Gr-PMMA layer (*t_Gr-PMMA_*) was measured using the scratch test method through atomic force microscopy (AFM) [21]. After etching the Cu foil, the layer was deposited on Si wafer and was scratched using a scalpel without damaging the substrate. AFM images of the scratch were acquired using a Dimension Icon (Bruker), in the Peak Force Tapping mode using ScanAsyst-Air probes (stiffness 0.2−0.8 N/m, frequency ∼80 kHz). The average depth of the scratch below the mean surface plane, corresponding to the film thickness, was evaluated using the cross-section analysis of the Nanoscope Analysis software. Several scratches were measured for each deposited layer to allow statistical analysis of data.

The final thickness of the produced Gr-PMMA nanolaminate was determined as the mean of 10 measurements with a digital micro-meter with a resolution of 0.1 μm (Mitutoyo, Kawasaki, Japan).

### 2.3. Raman Spectroscopy

Raman spectroscopic mapping has been performed on the nanolaminate with a Renishaw Invia Raman Spectrometer on an area of 100 × 100 μm^2^, by acquiring spectra at steps of 3 μm, using a 514 nm laser excitation and a 100× lens. Laser power on the specimens was kept below 1 mW to avoid laser-induced heating. Raman spectra were baseline corrected, and characteristic peaks of graphene were fitted to Lorentzian functions, the spectroscopic parameters (peak position and full width at half maximum, FWHM) being recorded at each position of the mapping.

### 2.4. Uniaxial Tensile Testing

Tensile tests on the nanolaminates were performed using a micro-tensile tester (MT-200, Deben Ltd., Woolpit, UK) equipped with a 5 N load cell. Rectangular specimens with a gauge length of 25 mm and a width of 1 mm were secured onto paper testing cards using a two-part cold curing epoxy resin. The specimens were then loaded in tension with a crosshead displacement speed of 0.2 mm min^−^^1^. Stress and strain were calculated based on the measured machine-recorded forces and displacements and, for each specimen, the Young’s modulus was estimated through a linear regression analysis of the initial linear portion of the stress–strain curves (up to ~0.4% strain). The estimation of both the Young’s modulus and tensile strength was obtained by the mean values of at least ten specimens, and the experimental errors are the deviation from the mean values.

### 2.5. Scanning Electron Microscopy

Scanning Electron Microscopy (ZEISS SUPRA 35VP SEΜ) was employed to assess the morphology of the produced nanolaminates.

### 2.6. Permeability Measurements

The permeabilities of oxygen and carbon dioxide through nanolaminate films were measured at different R.H. levels using a commercial permeabilimeter (Multiperm by ExtraSolutions S.R.L. Pisa, Italy). Both sides of the nanolaminate are exposed to gas streams at a total pressure of 1 atm, so that any convective contribution to the gas transport through the membrane can be disregarded and the whole measurement chamber is maintained at a controlled temperature (25 °C in the case at hand). To remove any low-molecular-weight compound possibly absorbed within the membrane, before each permeation test, both sides of the film are flushed with pure nitrogen (service gas) for a time span at least 5 times as long as the expected transient of the permeation experiment. Each permeation test starts by switching the gas stream on the upstream side from the service gas to the testing gas (CO_2_ or O_2_ in the case at hand) at a fixed R.H. Simultaneously, the same R.H. condition is also imposed on the downstream side for the service gas (nitrogen) flow, thus realizing symmetric R.H. conditions. Once the experiment is started, the service gas on the downstream side continuously removes the permeated test gas molecules. The concentration of test gas molecules within the service gas stream is measured in situ by using an electrochemical sensor in the case of oxygen and an infrared sensor in the case of carbon dioxide. Once steady state permeation conditions are attained (i.e., constant permeation flux through the membrane and, in turn, constant concentration of test gas within the service gas stream on the downstream side), the steady-state flux of the test gas through the membrane, *J_ss_*, is estimated. Then, from this value, the permeability coefficient, *P*, of the test gas is calculated according to the following equation:

(1) $$P=\frac{J_{SS}}{\left(\Delta p/t\right)}$$

where t represents the membrane thickness and ∆*p* stands for the difference of the partial pressure of the test gas between the upstream and the downstream sides. The partial pressure of the test gas on the downstream side is very close to 0. All these calculations are automatically performed by the software governing the apparatus. In the operating conditions of the experiments, since the nitrogen solubility is much smaller than those of oxygen, carbon dioxide and water [22,23,24,25], the effects of nitrogen on the measured permeabilities can be neglected. Tests were performed on 3.5 × 3.5 cm membranes; using adhesive Al masks (standard masks provided by the manufacturer of the permeabilimeter) to reduce the exposed surface, the actual area subjected to the flux was, however, a circle of 2.01 cm^2^.

## 3. Results and Discussion

### 3.1. Characterization of Materials

**Figure 1:**
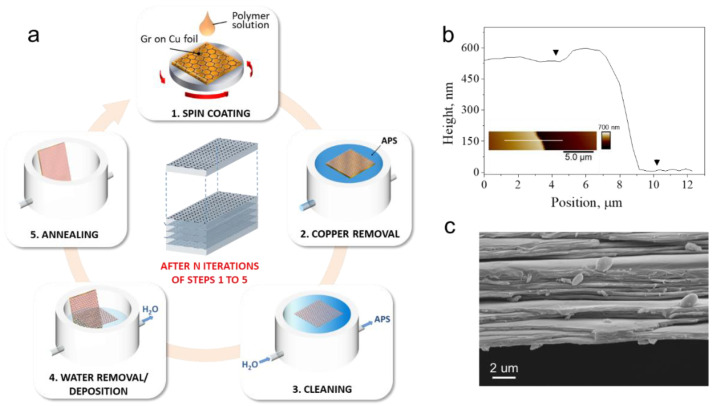
(**a**) Schematic illustration of the iterative ‘lift-off/float-on’ process combined with wet depositions adopted to produce the Gr-PMMA nanolaminates. (**b**) Thickness evaluation of the single Gr-PMMA layer deposited on a Si wafer: representative cross-section of the scratch and AFM image as inset. (**c**) SEM image in the cross-section plane of the nanolaminate.

By using the all-fluidic ‘lift-off/ float-on’ process schematically depicted in Figure 1a, a freestanding nanolaminate consisting of twenty graphene–PMMA (Gr-PMMA) layers was produced. In the nanolaminate configuration, the volume fraction of graphene (*V_Gr_*) is defined as:

(2) $$V_{Gr}=\frac{t_{Gr}}{t_{Gr}+t_{P}}$$

with $t_{Gr}\;$ being the thickness of monolayer graphene (0.334 nm) and $t_{P}$ the thickness of the polymeric layer. Therefore, to estimate the content of graphene in the produced nanolaminate, a preliminary investigation of the thickness of the single Gr-PMMA layer has been performed. According to the scratch test method, the thickness of the single Gr-PMMA layer (*t_Gr-PMMA_*) is ca. 550 nm (Figure 1b), indicating that the *t_PMMA_* ≅ 549.5 nm and that the nominal volume fraction of graphene in the nanolaminate was 0.06%. This value matches well the actual volume fraction, as inferred inversely from the final thickness of the nanolaminate, which was measured using a digital micro-meter. A representative SEM image of the cross-section of the specimen is shown in Figure 1c and clearly reveals the stratified architecture of the produced membrane, highlighting the regular lamination sequence of the Gr-PMMA layers.

**Figure 2:**
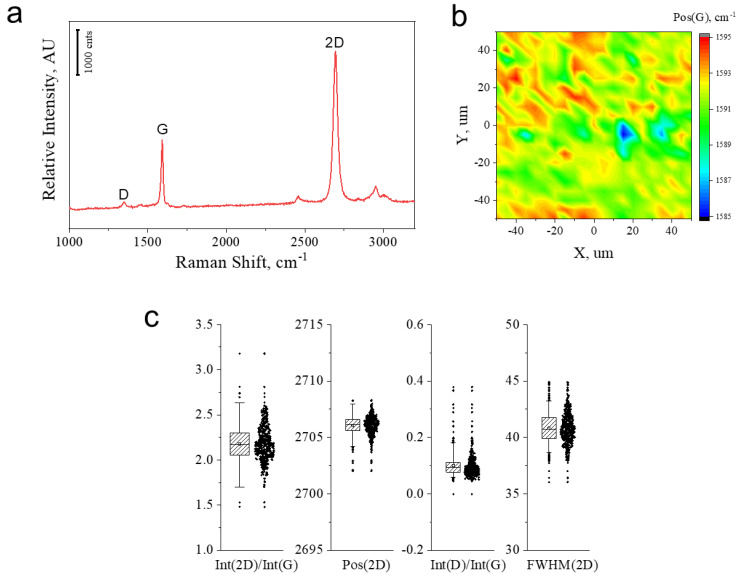
(**a**) Representative Raman spectra collected from the Gr-PMMA nanolaminate under investigation. (**b**) Contour map of the position of the G peak. (**c**) Box plots for Int(2D)/Int(G), Pos(2D), Int(D)/Int(G) and FWHM(2D).

Raman spectroscopy was employed to assess the quality of the graphene layers incorporated in the nanolaminate. The representative Raman spectrum acquired from the produced nanolaminate is shown in Figure 2a, and presents the typical spectroscopic features of graphene, namely the graphitic (G) and the strong second-order (2D) peaks [26]. The homogeneous distribution of these features within the specimen is revealed in the contour plots shown in Figure 2b, thus confirming the successful incorporation of continuous graphene into the nanolaminate. In particular, the G peak located at ~1592 cm^−1^ and the 2D peak located at ~2706 cm^−1^ reveal that the graphene experiences a slight residual compression. The Int(2D)/Int(G) ratio larger than 2 indicates the monolayer feature of graphene, which is retained after multiple depositions; however, the non-zero but small Int(D)/Int(G) indicates the presence of minor structural defects that are likely induced during the manipulation of the layers in the lamination process (Figure 2c).

**Figure 3:**
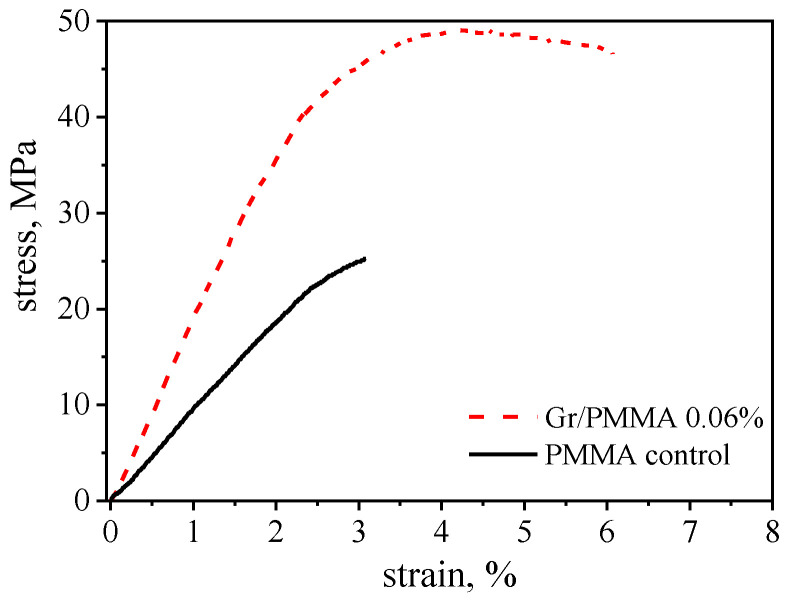
Representative stress–strain curve of the Gr-PMMA nanolaminate (red dotted line) and of the control PMMA sample (black solid line) obtained by uniaxial tensile testing.

The mechanical performance of the produced nanolaminate has been assessed by means of uniaxial tensile testing and representative stress strain curves for the Gr-PMMA nanolaminate, as well as for the PMMA control sample, are shown in Figure 3. It is interesting to note that the addition of graphene to the nanolaminate results in a substantial increase in the Young’s modulus, from 1.8 ± 0.2 MPa to 2.4 ± 0.2 MPa. Additionally, the tensile strength is found to increase by ca. 100% compared to the control sample. By following the approach adopted by Vlassiouk et al. [27], the use of a simple rule of mixture makes it possible to estimate the effective contribution of graphene to the modulus and the strength of the nanolaminate, which yield 1 TPa and 40 GPa, respectively. This unprecedented improvement in the mechanical properties under tension has previously been ascribed to the efficient reinforcement provided by the large-size graphene sheets in the nanolaminate architecture [20]. Furthermore, it is also worth noting that the nanolaminate shows a tougher behaviour, with a two-fold increase in elongation at break, compared to the neat PMMA. This can likely be ascribed to a possible phemonenon of slippage of the graphene layers upon large deformations, as previously observed by Liu et al. [28].

### 3.2. Results of Permeability Measurements

Permeability tests of gaseous CO_2_ and O_2_ through the nanolaminate films were performed at 25 °C at 1 atm. Experiments were conducted at three R.H. values (0, 50 and 80%), symmetrically imposed at upstream and downstream sides of the films.

**Figure 4:**
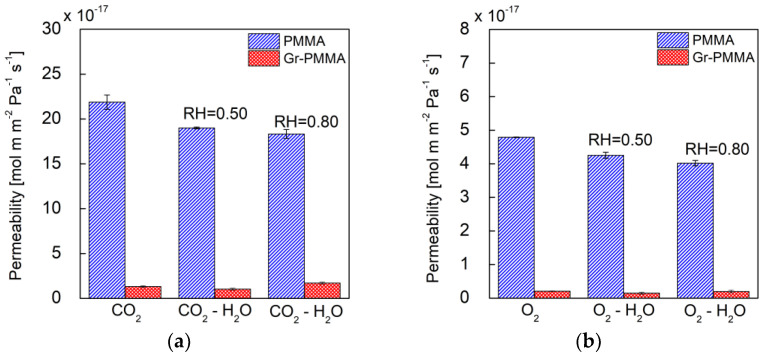
Gas permeability coefficients at 25 °C through PMMA (blue bars) and Gr-PMMA (red bars) for (**a**) CO_2_ and humidified CO_2_ and for (**b**) O_2_ and humidified O_2_.

In Figure 4a,b bar diagrams of the CO_2_ and O_2_ (pure and humidified) permeability coefficients for the pure PMMA nanolaminate and the Gr-PMMA nanolaminate membranes are presented. The corresponding numerical values are also reported in Table 1 and Table 2, respectively. We remark that the permeability coefficients of the pure gases in neat PMMA nanolaminate agree with the values reported in the literature for commercial amorphous PMMA [22,23,29,30]. In particular, Min et al. [22] found a value of permeability coefficients at 35 °C equal to 0.4 Barrer for CO_2_ and equal to 0.11 Barrer for O_2_; Raymond et al. [23] found a value of *P* at 35 °C equal to 0.6 Barrer for CO_2_ and equal to 0.1 Barrer for O_2_ $\left(1\;\mathrm{Barrer}=10^{-10}\frac{{\mathrm{cm}}_{STP}^{3}\;\mathrm{cm}}{{\mathrm{cm}}^{2}\;s\;\mathrm{cmHG}}\right)$. This indicates that the lamination process does not, per se, induce relevant effects on the bulk structure of PMMA layers within the nanolaminate. The enhanced CO_2_ and O_2_ barrier properties measured in the whole range of investigated R.H.s and discussed below are mainly to be ascribed to the addition of graphene layers, since the morphological investigations performed on the nanocomposite showed that the bulk of the PMMA layers did not exhibit evident orientation effects or crystallization effects induced by the contact between macromolecules and graphene layers, which may affect the value of the permeability coefficient [31].

Several literature contributions report an improvement in the barrier properties of PMMA after the incorporation of GRM in the form of flakes/nanosheets [7]. For instance, Morimune et al. observed a significant improvement in barrier properties in discontinuous PMMA/GO nanocomposites following the addition of 1 wt% (0.5 vol%) of μm-size GO to the PMMA matrix, leading to a decrease in the O_2_ permeability by 50% [32]. Chang et al. prepared PMMA nanocomposites filled with carboxyl–graphene nanosheets and found that, compared to neat PMMA, the nanocomposite membranes with 0.5 wt% (0.25 vol%) carboxyl–graphene nanosheets loading showed about 70% and 61% reductions in O_2_ and H_2_O permeability, respectively [33].

Indeed, the nanolaminate investigated here shows a higher reduction in the O_2_ permeability when compared to the neat PMMA (ca. 95%), which was achieved with the addition of only 0.06 vol% of graphene. This enhancement of the barrier properties is to be ascribed to the controlled and homogeneous dispersion of the graphene layers with high aspect ratio within the PMMA matrix, which was achieved within the alternate-layered structure of the nanolaminate. In fact, it has been theoretically estimated and experimentally demonstrated that, when graphene sheets possess an aspect ratio higher than 1000, a tiny volume fraction of graphene makes it possible to achieve a significantly improved barrier performance (P_nanocomposite_/P_polymer_ ≅ 0.1) [31]. Based on the Nielsen approximation [10] for polymer nanocomposites with layered 2D sheets oriented perpendicularly to the diffusion direction, when the aspect ratio approaches infinity (as for the cm-size, atomic-thick graphene used in the nanolaminate at hand), the amount of the filler materials required to achieve good barrier properties is vanishingly small.

It is important to underline, however, that large-scale graphene monolayers produced via CVD contain numerous defects (e.g., vacancies, grain boundaries, wrinkles) that originate during the synthesis and the transfer/deposition processes, and therefore its barrier properties are lower than those of pristine graphene [34]. It has therefore been suggested that permeation through defects can be mitigated by using more layers of graphene and/or nanolaminate structures allowing synergistic effects between different materials, such as the graphene/alumine-oxide nanolaminates proposed by Sagade et al. [35].

In view of these observations, a reasonable physical picture for the permeation process of the penetrant molecules through the polymer–graphene-based multilayer nanolaminate systems consists in assuming a series mechanism involving the ordinary diffusive behaviour within the polymer-penetrant phase and diffusive jumps through the graphene defects (voids, cracks) statistically distributed onto each graphene monolayer within the composite [8,9,36]. According to this framework, the permeability coefficient of each penetrant through the nanolaminate membrane, *P_Gr-PMMA_*, can be expressed by the following equation [8,9,37]:

(3) tGr−PMMAPGr−PMMA=∑i=1NtGrPi,Gr+∑j=1MtPMMAPj,PMMA=N×tGrPGr+M×tPMMAPPMMA

where *t_Gr_* and *t_PMMA_* represent the single graphene layer and the single PMMA layer thickness, respectively, and *N* and *M* stand for the total numbers of graphene and PMMA layers forming the nanolaminate, respectively (in our case, *N* = *M* = 20). Finally, *P_i,Gr_* and *P_j,PMMA_* stand for the testing gas permeability coefficient through the *i*-th and *j*-th graphene and PMMA layer, respectively. Due to the low values of penetrant concentration expected to occur within the polymeric phases [25], one can reasonably assume that *P_j,PMMA_* does not depend significantly upon the position of the specific *j*-th layer. Moreover, when the number of PMMA/Gr layer sandwiches is sufficiently high to allow a reliable statistically “averaged” approach, one can also assume a unique “average” value for *P_i,Gr_* within the system, so that the second equality in Equation (3) holds true. To better elucidate the role of the distribution of the defect sizes present in the graphene layers on the permeation mechanism, one can start to analyse the case of the two pure gases investigated. In this regard, Equation (3) can be used to estimate the “average” permeability, *P_Gr_*, of each pure gas through the graphene layers within the nanocomposite. In this regard, it is worth noting that in the framework of Equation (3), under the reasonable assumption that the PMMA laminate behaves as the pure PMMA bulk phase, the permeation of the graphene layers includes the effect of the PMMA–graphene interphase. Therefore, *P_Gr_* must properly be considered the “average” permeability of the single graphene layer within the composite, which is in principle different from the value one would observe in the case of a hypothetical equivalent isolated graphene layer with the same “average” defect distribution. In fact, given the number of graphene and PMMA layers, the gas permeability through the Gr-PMMA nanocomposite and through the pure PMMA nanolaminates (see Table 1 and Table 2), *P_Gr_* can easily be calculated using the following expression:

(4) $$P_{Gr}=\frac{N\times t_{Gr}}{\frac{t_{Gr-PMMA}}{P_{Gr-PMMA}}-\frac{M\times t_{PMMA}}{P_{PMMA}}}$$

One obtains a value of *P_Gr_* equal to 8.53 × 10^−21^ [mol m m^−2^ Pa^−1^ s^−1^] in the case of CO_2_ and to 1.33 × 10^−21^ [mol m m^−2^ Pa^−1^ s^−1^] in the case of O_2_. The ratio between the pure CO_2_ permeability of a graphene layer and that of pure O_2_ is higher than its value for pure PMMA (6.40 vs. 4.57), thus suggesting that the presence of the graphene layers introduces a molecular ‘sieving’ mechanism in the nanocomposite ruled by the graphene layer defect size; this is not present in the glassy PMMA, which is characterized by solubility selectivity more than by size selectivity [36], in view of its high free volume. To support this hypothesis, it can be observed that the values of the kinetic diameters taken from the literature [38] are equal to 0.330 nm for CO_2_ and 0.346 nm for O_2_, which are in line with the higher *P_Gr_* observed for CO_2_.

In general, the gas permeability coefficient of a multilayer membrane, *P*, can be properly quantified according to the following phenomenological equation, as has already been adopted by Pierleoni et. al. [9] in dealing with pure gas permeabilities through differentGO-polymeric multilayer nanocomposite membranes:

(5) $$P=a\cdot e^{-b\cdot k_{d}}$$

where *k_d_* represents the kinetic diameter [9,38], and the parameter *b* is simply retrieved from the slope of the corresponding permeability coefficients curve (semi-log scale) versus the corresponding molecular kinetic diameter and provides a quantitative estimation of the “membrane sieving capacity”.

In this context, it is of interest to compare the values of *b* for pure PMMA and for Gr-PMMA nanolaminates in the case of CO_2_ and O_2_ with an “average” value of *b* for a single graphene layer. The corresponding values of *b* are about 95 nm^−1^ in the case of pure PMMA and about 115 nm^−1^ in the case of the PMMA–graphene nanolaminate and of the single graphene layer within the nanocomposite. Indeed, the value of *b* in the case of the nanocomposite is higher than that of the nanolaminate made of neat PMMA and slightly lower than the “average” *b* of the single graphene layer. This result is consistent with the described physical picture, pointing out that the ‘necking factor’ for the permeation mechanism of the penetrants is given by jumps through voids of proper size and shape distributed onto each graphene monolayer.

The stronger dependence of permeability on the molecular size of the penetrant compared to the pure PMMA nanolaminate membrane that was observed in the case of the single graphene monolayer, and hence for the nanocomposite, suggests that most graphene layers will mainly display nano-sized defects.

The role of graphene defects on the penetrant permeation mechanism in the nanocomposite system is expected to be significant also in the case of gas permeation in the presence of water vapour. To elucidate possible effects of water molecules on the mass transport mechanism of CO_2_ and O_2_, we have investigated the permeation of the two gases at different R.H. levels.

We first analysed the case of the pure PMMA nanolaminate film, finding that both the CO_2_ and O_2_ permeability coefficients decreased as a function of R.H. level. Starting from the analysis of pure gases, the steady-state permeation coefficient *P* can be expressed, in principle, as $P=\bar{D}\times \bar{S}$, where $\bar{D}$ and $\bar{S}$ represent, respectively, an average thermodynamic diffusion coefficient and an average solubility coefficient [37,39]. In fact, both diffusivity and solubility coefficients can be a function of penetrant concentration [39], and hence of the spatial position along the film thickness. In the case at hand, in the range of pressure considered, both the diffusivity and solubility coefficients of CO_2_ and O_2_ can be assumed to be constant.

In the case of humidified streams, water vapour is absorbed within the PMMA membrane, thus affecting both the diffusivity and solubility of the test gas. In particular, a monotonic decrease in the permeabilities of both gases with R.H. was observed. The prominent effect was expected to be the reduction of solubility of the gases in view of the higher condensability of water. In fact, water competes with permanent gases for adsorption on the excess free volume micro-voids characteristic of a glassy polymer [40].

In contrast, in the case of Gr-PMMA nanolaminate, a non-monotonic effect of R.H. on test gas permeabilities was detected. In fact, the experimentally determined permeabilities of both CO_2_ and O_2_ display, at the highest R.H. levels investigated, a value higher than that determined at R.H. = 0.5. Although lack of detailed information on the structure of graphene nanocomposites prevents a robust explanation of this effect, in the following we propose a reasonable interpretation of the observed behaviour.

In fact, the transport mechanism within the nanocomposite of each testing gas through each PMMA bulk phase is expected to follow the same monotonic decreasing trend as a function of R.H., observed for the pure PMMA nanolaminate. In addition, it is expected that water molecules compete with the testing gas molecules in the passage through the single-layer graphene nanocavities. Additionally, this effect in principle contributes to a decrease in the permeability of the testing gas through the overall nanocomposite. On these bases, the observed increase in *P* at higher R.H. levels could be explained by invoking the fact that the adsorption of water molecules locally affects the mass transport mechanism within the PMMA–graphene interface regions. In fact, it is well known that the CVD graphene layers within the PMMA–graphene nanolaminate may display a wrinkled morphology at the nanoscale [41] as an intrinsic consequence of the preparation procedure, which could be associated with the formation of micro-voids of nanoscale size in the interface regions In the literature, it has been found that in PMMA–graphene systems with similar wrinkled morphology [42] the adsorption of water induces an enlargement of such winkles, which would promote a further increase in the micro-void sizes within the interphase. This water-induced alteration of interphase structure would facilitate the in-plane diffusion of the testing gas along the graphene layers, which represents a necessary step for the permeating molecules to find an available nanochannel to pass through the graphene layer. Based on this physical picture, the increase in testing gas permeability coefficient at high R.H. is likely to be due to the interplay between the water-induced hindering of passageways through the graphene layers, the described decrease in the PMMA–graphene interphase density, and the well-established [39] decrease in the permeability coefficient of the testing gas as a function of relative humidity in a pure glassy polymer.

To isolate the average contribution of a single Graphene layer within the nanocomposite, we estimated its “equivalent” permeabilities *P_Gr_* by using Equation (4) as applied to the case of R.H. = 0.5 and R.H. = 0.8. The results are reported in Table 3. Quite interestingly, both CO_2_ and O_2_ “equivalent” average permeabilities for a single graphene layer also display such a non-monotonic behaviour. This result is to be expected since the “equivalent” graphene layer average permeability coefficient takes into account both the molecular jump mechanism through the graphene defects and the molecular transport mechanism within the interphase regions, so that a non-monotonic decrease in *P_Gr_* as a function of R.H. (and in particular an increase in it in the high R.H. value range) could result from the trade-off between the two different dependence of the described transport mechanism as a function of relative humidity.

## 4. Conclusions

Films with a nanolaminate architecture, made of controlled alternating PMMA layers and cm-size graphene monolayers, were produced via Chemical Vapour Deposition and characterized for their mechanical and gas barrier properties. A significant improvement of mechanical properties as compared to pure PMMA was registered, with enhanced stiffness, strength, and toughness, with the addition of only 0.06 vol% of graphene.

Oxygen and carbon dioxide permeability measurements, conducted at different R.H. levels, revealed that the addition of graphene monolayers led to a significant improvement in the gas barrier properties compared to neat PMMA, outperforming the traditional discontinuous graphene–particle composites, while using a similar filler content. A size-sieving effect of the graphene monolayer was evidenced, and an interesting effect of relative humidity on permeation properties was highlighted. In fact, a non-monotonic decrease behaviour of the gas permeability through the nanocomposite as a function of R.H. was found, different from the observed behaviour of pure nanolaminate PMMA, that has been tentatively attributed to the interplay between water-induced hindering of passageways through the graphene layers, the water-induced decrease in the PMMA–graphene interphase density associated with the enlarging of graphene layers wrinkles, and the decrease in the permeability coefficient of the testing gas as a function of relative humidity within the bulk polymer phase. Indeed, the latter is due to a well-established competitive mechanism of water and testing gas sorption within the polymer bulk phase.

## Figures and Tables

**Table 1:** Permeability coefficients of CO_2_ through the nanolaminates at different R.H. levels.

Nanolaminate/*Permeating Gas*	*P* [mol∙m∙m^−2^∙Pa^−1^∙s^−1^]	*P* [Barrer]
PMMA/*CO*_2_	21.9 (±0.8) × 10^−17^	6.5 (±0.2) × 10^−1^
Gr-PMMA/*CO*_2_	1.30 (±0.1) × 10^−17^	0.39 (±0.03) × 10^−1^
PMMA/*CO*_2_-*H*_2_*O @RH* = 0.50	19.0 (±0.1) × 10^−17^	5.67 (±0.03) × 10^−1^
Gr-PMMA/*CO*_2_-*H*_2_*O @RH* = 0.50	1.0 (±0.1) × 10^−17^	0.30 (±0.03) × 10^−1^
PMMA/*CO*_2_-*H*_2_*O @RH* = 0.80	18.3 (±0.5) × 10^−17^	5.5 (±0.2) × 10^−1^
Gr-PMMA/*CO*_2_-*H*_2_*O @RH* = 0.80	1.7 (±0.1) × 10^−17^	0.51 (±0.03) × 10^−1^

**Table 2:** Permeability coefficients of O_2_ through the nanolaminates at different R.H. levels.

Nanolaminate/*Permeating Gas*	*P* [mol∙m∙m^−2^∙Pa^−1^∙s^−1^]	*P* [Barrer]
PMMA/*O*_2_	4.79 (±0.01) × 10^−17^	1.434 (±0.003) × 10^−1^
Gr-PMMA/*O*_2_	0.21 (±0.01) × 10^−17^	0.063 (±0.003) × 10^−1^
PMMA/*O*_2_-*H*_2_*O @RH* = 0.50	4.25 (±0.09) × 10^−17^	1.27 (±0.03) × 10^−1^
Gr-PMMA/*O*_2_-*H*_2_*O @RH* = 0.50	0.15 (±0.03) × 10^−17^	0.045 (±0.009) × 10^−1^
PMMA/*O*_2_-*H*_2_O @RH = 0.80	4.02 (±0.08) × 10^−17^	1.20 (±0.02) × 10^−1^
Gr-PMMA/*O*_2_-*H*_2_*O @RH* = 0.80	0.20 (±0.04) × 10^−17^	0.06 (±0.01) × 10^−1^

**Table 3:** Estimated permeability coefficients of CO_2_ and O_2_ through a graphene nanolayer at different R.H. levels.

Permeating Gas	*P* [mol∙m∙m^−2^∙Pa^−1^∙s^−1^]	*P* [Barrer]
CO_2_	8.5 (±0.8) × 10^−21^	2.5 (±0.2) × 10^−5^
CO_2_ @ RH = 0.5	6.6 (±0.9) × 10^−21^	2.1 (±0.3) × 10^−5^
CO_2_ @ RH = 0.8	11.0 (±0.9) × 10^−21^	3.3 (±0.3) × 10^−5^
O_2_	1.3 (±0.1) × 10^−21^	0.40 (±0.03) × 10^−5^
O_2_ @ RH = 0.5	1.0 (±0.2) × 10^−21^	0.28 (±0.06) × 10^−5^
O_2_ @ RH = 0.8	1.3 (±0.2) × 10^−21^	0.38 (±0.06) × 10^−5^

## Data Availability

All data supporting reported results are reported in the manuscript.
